# Notes and Notices

**Published:** 1897-11

**Authors:** 


### NOTES AND NOTICES.

Dr. James' Sanitarium.—The Institution opened by Dr. Bushrod W. James, October 5th, 1886, and now located at 1717 Green Street, has been renovated during the summer, and is ready to receive a limited number of neurasthenic or rest-cure patients, as well as the usual eye, ear, and general surgical cases for operation and treatment. The price of rooms varies according to location. Massage, medical electricity, or vapor baths administered, should the case require it.

Chopcheenee φ in Syphilis.—This is a plant indigenous to India and other Eastern countries. Some Indian medical writers are of the opinion that this medicine was introduced into India by Chinese traders; however it be, this much is certain, that this medicine has been in use in India for a very long time, as we find evidenced by medical works written some three hundred years ago. The root is the only part used as medicine. It is said to be useful in rheumatism, epilepsy, insanity, and particularly syphilis. It is a most renowned medicine for syphilis in India. Its action is more decided in the secondary stage, especially when the skin and mucous membrane are the seat of suffering. It is a non-poisonous plant, and so its action is mild and not so violent as that of Mercury, still it is in no way inferior to the latter in its efficacy in syphilis when the disease has gone to the secondary stage. The action of the medicine is generally known within one to three weeks. It should be continued for a sufficiently long time, according to the severity of the symptoms and chronicity of the case, three times a day. Chopcheenee φ can be had of Bœricke, Runyon & Ernesty, 497 Fifth Avenue, New York.

Dr. Annie Lowe Geddes will remove, December 1st, to 69 Fullerton Avenue, North, Montclair, N. J.

The Transactions of the International Hahnemannian Association for the meeting held in June, 1897, have now been printed and will be shortly issued. A copy will be sent to all members not in arrears for annual dues. Every member should see to it that his dues are paid and secure a copy of these valuable records.

### DEATH FROM VACCINATION.

*Editor of Homœopathic Envoy:*

Your articles on vaccination have greatly interested me, especially as two neighbors' children have suffered such ill effects from "compulsory vaccination."

With one little chap "it did not work," so the teacher of the public school he attended took him herself two weeks after the first vaccination, and insisted upon its being done "strong," the child in consequence nearly losing his arm, and being still in a very weak condition—this after a little over one year. The second case, a child of our alderman, ——— ———, died soon after vaccination, the symptoms being too startling to allow a public funeral.

A Subscriber.

*Syracuse, N. Y., Nov. 6th, 1897.*

Antimonium-Tartaricum in Whooping Cough.—The child is irritable, cross, and cries when approached; coughs whenever it gets angry or after eating, the cough culminates in the vomiting of food and mucus, much rattling of mucus in chest without much expectoration. Coughing and gaping consecutively, tongue coated white, thick pasty coating.—*Hom. Jour. Obstetrics.*

Ammonium-Carbonicum in Scarlet Fever.—Stoppage of the nose at night, child must breathe through the mouth; long-lasting coryza. The child's nose is stopped; it starts from sleep; nose bleeds when washing face or hands; tonsils swollen, bluish, covered with offensive mucus; tendency to gangrenous ulceration of tonsils, eruption bright red, miliary rash. Is indicated where rash recedes too early and paralysis of the brain threatens. Malignant cases with stupor, starting from sleep, putrid sore throat, saliva adhesive, swelling of parotid and cervical glands, stertorous breathing, excessive vomiting, and involuntary stools.—*Hom. Jour. Obstetrics.*

---

### FUN FOR DOCTORS.

A Prudent Doctor.—Patient: "I don't suppose you are particular whether I pay you now or settle in full when you get through with me?"

Doctor.—"Perhaps you'd better pay now. I would be quite willing, as you say, to wait until I get through with you, but the fact is, your will might be contested, you know, and I might get nothing at all."

An Esteemed Contemporary.—"Doctor," said the dying editor, "I have one last favor to ask you."

"Name it," said the doctor.

"I want you to attend the editor of the other paper."

Cheaper.—Mrs. Cumso (newspaper in hand): "A movement is on foot to make drugs cheaper."

Cumso: "Good enough! That will bring sickness within reach of all."

Absent.—Daughter: "Shall we invite Dr. Bigfee to the reception?"

Mother: "I think we'd better not, he's so absent-minded. He might charge it in his bill."

Medical Ethics.—"Doctor, my little boy is in a very critical state, and I am satisfied that Dr. Probe, who is now attending him, doesn't understand the case. I wish you would come right over and see the boy."

"I don't see how I can do it. Probe and I were old friends, and in these matters of professional courtesy we have to be mighty careful."

"But great heavens, man! if you don't come the boy may die."

"That's just the point. Suppose I should save the boy. Why, Probe would never forgive me."

There was a young woman, named Margery,
Whose head was a walking mena(*r*)gerie;
They said, "You should wash,"
But she answered, "Oh, bosh!
I'll apply some Unguentum Hydragari."

